# Synthetic Phase-Sensitive Inversion Recovery and Extramural Vascular Invasion Scoring Systems for Vascular Invasion Detection and Metastasis Risk in Rectal Cancer

**DOI:** 10.1148/rycan.250564

**Published:** 2026-09-18

**Authors:** Yuxi Ge, Xiaofei Wu, Heng Zhang, Zi Wang, Kang Wang, Wenjie Chai, Wenbo Xu, Jiankun Dai, Yutian Zhao, Shudong Hu

**Affiliations:** ^1^Department of Radiology, Affiliated Hospital of Jiangnan University, 1000 Hefeng Road, Wuxi, Jiangsu, China 214000; ^2^Department of Radiology, Donghai County People’s Hospital, Lianyungang, Jiangsu, China; ^3^Fudan University Wuxi Research Institute, Wuxi, Jiangsu, China; ^4^GE Healthcare, MR Research China, Beijing, China; ^5^Department of Oncology, Affiliated Hospital of Jiangnan University, Wuxi, Jiangsu, China

**Keywords:** Abdomen/GI, MR-Imaging, Diagnosis, Prognosis & Prediction

## Abstract

**Purpose:**

To develop and validate a modified extramural vascular invasion (EMVI) scoring system with synthetic phase-sensitive inversion recovery (SyPSIR) images to improve vascular invasion detection and metastasis prediction in patients with rectal cancer.

**Materials and Methods:**

Participants with rectal cancer were eligible for this single-center prospective study between October 2020 and June 2024, with follow-up intervals of 3–6 months (median follow-up, 23 months; range, 3–50 months). All participants underwent EMVI evaluation using conventional T2-weighted imaging–based and modified synthetic phase-sensitive inversion recovery–incorporated scoring systems, with diagnostic performance for pathologic EMVI detection evaluated against histopathology. Receiver operating characteristic curve analysis was used to evaluate the diagnostic performance of the pathologic EMVI results. Kaplan-Meier survival analysis and multivariable Cox regression analysis were used to compare the prognostic performance of the scoring systems, and a nomogram for metastasis risk stratification was developed.

**Results:**

This study included 356 participants (mean age, 65 years ± 10 [SD]; 238 males) with rectal cancer (304 with pathologically confirmed EMVI). The modified EMVI scoring system demonstrated better diagnostic performance for pathologic EMVI detection than the conventional T2-weighted imaging system (sensitivity: 59%–80% vs 33%–49%; area under the receiver operating characteristic curve: 0.72–0.80 vs 0.59–0.68; *P* < .05). Multivariable Cox regression analysis confirmed that a modified MRI-based EMVI (mrEMVI) was the most independent predictor of metastasis-free survival (MFS) (hazard ratio, 10.76; *P* < .001), with Kaplan-Meier analysis demonstrating its better prognostic stratification ability in both the training and validation cohorts. A prognostic nomogram incorporating the modified mrEMVI, mucinous component, mrN stage, mrT stage, and tumor deposit achieved excellent discrimination for 3-year MFS (area under the receiver operating characteristic curve, 0.86–0.98), with reasonable calibration, and demonstrated clinical utility across relevant decision thresholds.

**Conclusion:**

The modified EMVI scoring system outperformed the conventional T2-weighted imaging system in both pathologic EMVI detection and metastasis prediction. The integrated nomogram incorporating the modified mrEMVI provides a robust tool for accurate 3-year MFS prediction.

**Keywords:** Abdomen/GI, MR-Imaging, Diagnosis, Prognosis & Prediction

*Supplemental material is available for this article.*

© The Author(s) 2026. Published by the Radiological Society of North America under CC BY 4.0 license.

## Graphical abstract

**Figure:**
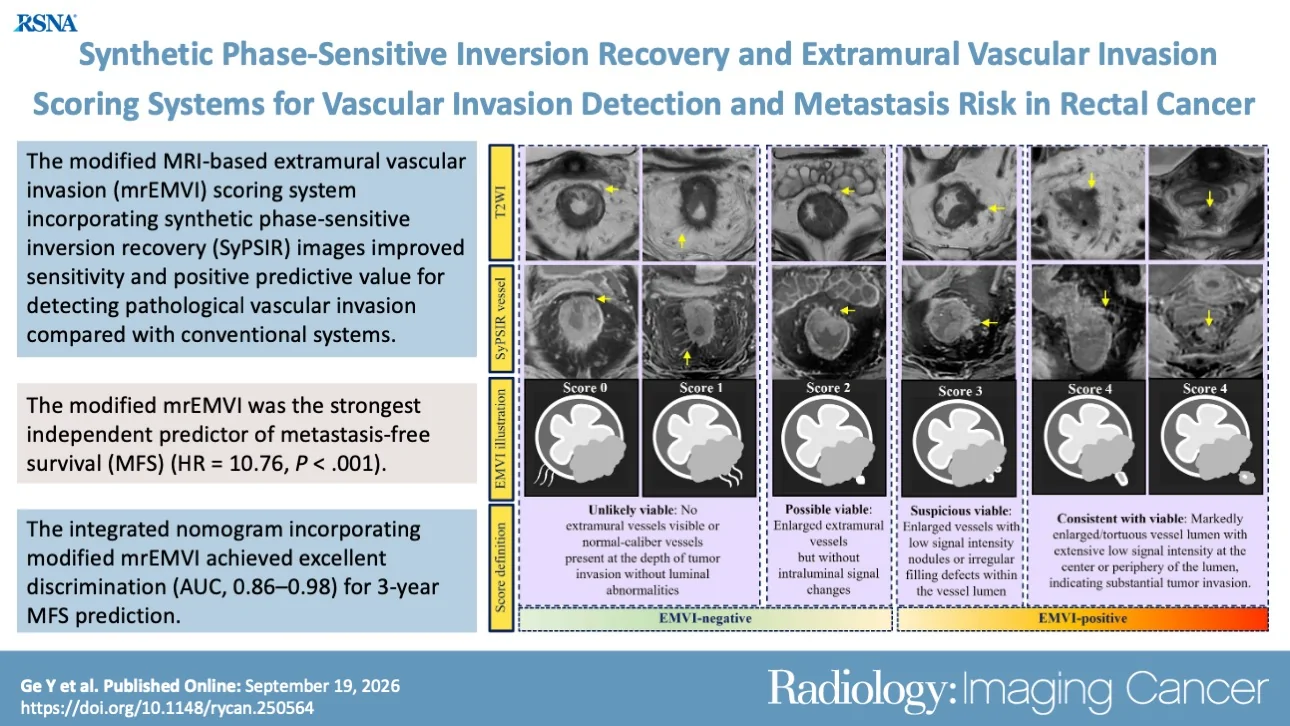

**Summary**

An MRI-based extramural vascular invasion scoring system using conventional T2-weighted imaging and synthetic phase-sensitive inversion recovery images improved detection of vascular invasion and predicted distant metastasis risk in patients with rectal cancer.

**Key Points**

■ Compared with conventional systems, the incorporation of synthetic phase-sensitive inversion recovery (SyPSIR) images into the extramural vascular invasion (EMVI) scoring system increased the sensitivity (59%–80% vs 33%–49%) and positive predictive value (40%–52% vs 33%–39%) for detecting pathologic vascular invasion.
■ The modified MRI-based EMVI (mrEMVI) was the strongest independent predictor of metastasis-free survival (MFS) (hazard ratio, 10.76; *P* < .001).
■ The integrated nomogram incorporating modified mrEMVI achieved excellent discrimination (area under the receiver operating characteristic curve, 0.86–0.98) for 3-year MFS prediction.

## Introduction

In rectal cancer, patient prognosis is determined primarily by tumor and nodal stage, surgical resection margins, and other pathologic features, such as venous invasion, perineural invasion, and tumor differentiation (1,2). Notably, extramural venous invasion (EMVI) is a well-established independent adverse prognostic factor (3). The presence of EMVI is associated with substantially increased rates of local recurrence, distant metastasis, and lymph node involvement and decreased overall survival (4–6). MRI is the standard method for preoperative local staging and has demonstrated considerable utility in identifying EMVI. Compared with TN staging, evaluation of MRI-based EMVI (mrEMVI) provides superior prognostic stratification (7,8). Therefore, mrEMVI is associated with high-risk disease characteristics and represents a critical adverse prognostic factor for risk stratification, supporting the consideration to increase the intensity of neoadjuvant treatment (9). Consequently, accurate identification of EMVI on preoperative MR images is essential for optimal risk stratification and individualized treatment planning.

Smith et al (10) were the first to assess EMVI on MR images by developing a standardized five-point scoring system (scores 0–4) for T2-weighted imaging sequences, demonstrating its histopathologic correlation in patients with rectal cancer. However, T2-weighted imaging–based EMVI assessment has shown suboptimal diagnostic performance, with a sensitivity ranging from 36% to 61% (1,11,12). Advanced imaging approaches have shown modest improvements, with diffusion-weighted imaging achieving 55% sensitivity despite improved contrast (13), dynamic contrast-enhanced texture analysis reaching 67% but requiring contrast agents (14), and intravoxel incoherent motion and diffusion kurtosis imaging improving to 68% with lengthy acquisitions (15). Deep learning methods plateaued at 55%–63% despite large training datasets (16), and recent deep learning reconstruction–based MRI showed no significant improvement in EMVI detection despite enhanced image quality (17). These persistent suboptimal sensitivities highlight the critical need for novel imaging approaches to enhance EMVI detection in rectal cancer.

Synthetic MRI is an emerging quantitative imaging technique characterized by its ability to rapidly and simultaneously acquire T1-weighted, T2-weighted, and proton density maps that are intrinsically spatially co-registered (18,19). On the basis of these maps, various contrast-weighted images can be synthesized, such as T1-weighted, T2-weighted, and phase-sensitive inversion recovery images. Wang et al (20) reported that synthetic phase-sensitive inversion recovery (SyPSIR) images can enhance the visualization of small vessels in the brains of patients experiencing ischemic strokes by leveraging the positive phase characteristics of slow-flowing blood. The earlier preliminary study revealed that compared with conventional T2-weighted imaging, SyPSIR images may enhance vessel–tumor contrast, which improved EMVI detection sensitivity from 40%–49% to 77%–86% for different readers in 106 patients with rectal cancer (21). However, SyPSIR images have not yet been incorporated into EMVI scoring systems, and the predictive value of a modified mrEMVI scoring system for metachronous distant metastasis remains unclear.

In this study, we hypothesized that enhanced vascular visualization using SyPSIR images would improve risk stratification in patients with rectal cancer. This study aimed to establish a modified mrEMVI scoring system by incorporating SyPSIR images into an existing system and to investigate the performance of this modified system for predicting metachronous distant metastasis in patients with locally advanced rectal cancer.

## Materials and Methods

### Patients

Between October 2020 and June 2024, consecutive patients with rectal cancer were prospectively enrolled in this study. This prospective study was approved by the institutional review board and ethics committee of Affiliated Hospital of Jiangnan University (LS2020145). All participants provided written informed consent before enrollment. The inclusion criteria were as follows: *(a)* histologically confirmed primary rectal cancer diagnosed between October 2020 and June 2024; *(b)* availability of pretreatment MRI scans, including synthetic MRI; *(c)* pretreatment MRI indicating locally advanced disease (T2N positive or T3-4N0-2); and *(d)* completion of total mesorectal excision or a neoadjuvant chemoradiotherapy regimen. The exclusion criteria were as follows: *(a)* clinically staged T1/2N0M0 rectal cancer; *(b)* metastatic disease at initial presentation (M1 stage) or recurrent rectal cancer; *(c)* prior antitumor therapy administered before baseline MRI; *(d)* uninterpretable pretreatment MRI; *(e)* image quality insufficient for confident EMVI assessment; *(f)* no inpatient management after MRI staging; *(g)* incomplete neoadjuvant therapy course; *(h)* loss to follow-up within the first posttherapy; or *(i)* nonadenocarcinoma histopathology confirmed postoperatively.

A subset of 106 participants included in this study was previously reported in a prior analysis that focused on the quality of SyPSIR images and their additional diagnostic value for EMVI evaluation when combined with T2-weighted imaging, using histopathology as the reference standard (21). However, a modified mrEMVI scoring system incorporating SyPSIR sequences was not developed, and the prognostic utility of such a system in predicting metachronous distant metastasis was not evaluated.

### MRI Acquisition

All MRI examinations were performed with a 3.0-T scanner (Signa Architect; GE HealthCare) using a 16-channel phased-array body coil. High-resolution T2-weighted images were acquired in axial, sagittal, and coronal orientations with a repetition time of 3227–5326 msec, an echo time of 102 msec, a field of view of 24 × 24 cm, a matrix size of 320 × 224, a section thickness/gap of 3/0.3 mm, 18–28 sections, and 1 excitation. Synthetic MR images were obtained in the axial plane using the vendor-provided commercially available MAGiC sequence (GE HealthCare). The imaging parameters were as follows: a repetition time of 4000 msec; an echo time_1_/echo time_2_ of 19.5/97.3 msec; saturation delay times of 210, 610, 1810, and 3810 msec; a field of view of 24 × 24 cm; a matrix size of 320 × 224; a section thickness/gap of 3/0.3 mm; 18–28 sections; and an acquisition time of 4 minutes and 32 seconds. T1-weighted, T2-weighted, and proton density maps were fitted from the acquired MAGiC data and used to generate SyPSIR images via S_PSIR_ = proton density × e^−echo time/T2^×(1−2e^−inversion time/T1^+e^−repetition time/T1^), where the repetition time is 8000 msec, the echo time is 10 msec, and the inversion time is 10 msec according to the previous study (21). The quantitative maps were fitted, and the SyPSIR images were generated using Synthetic MR software (version 8.0; GE HealthCare). The detailed imaging parameters for all the sequences are summarized in Table S1. The image quality and tumor-to-vessel signal intensity of the SyPSIR images were analyzed as described in Appendix S1.

### Radiologic Evaluation

Three radiologists (S.H., Z.W., and K.W.; 22, 12, and 5 years of experience, respectively, in abdominal MRI) who were blinded to the clinical and pathologic data independently reviewed the anonymized and randomized MR scans for EMVI assessment. Each radiologist evaluated conventional T2-weighted imaging and SyPSIR images extracted from the same MRI examination. To minimize recall bias, a washout period of 4 weeks was maintained between the evaluation of the two image sets, with the order of image set review randomized for each radiologist. To assess intraobserver agreement, radiologist 1 (S.H., 22 years of experience) re-evaluated all the images 4 weeks after the initial assessment and was blinded to the initial readings and clinical outcomes. When MRI-based EMVI was identified, its location was recorded according to three anatomic regions: the superior rectal vein territory (superior rectal vein trunk or tributaries within 2 cm proximally), the superior rectal vein tributary zone (left/right tributaries), and the peritumoral region (≤1 cm beyond the deepest tumor invasion). Additional imaging parameters, including the mucinous component, tumor longitudinal length, circumferential extent, mrT stage, mrN stage, tumor deposits at MRI, and mesorectal fascia involvement status, were independently evaluated by two radiologists (Z.W. and K.W.). Any discrepancies were resolved by a third senior abdominal radiologist to reach a final consensus (S.H.).

### mrEMVI Scoring System

The existing mrEMVI scoring system is based on T2-weighted imaging sequences, and the score ranges from 0 to 4, with 0 indicating no peritumoral vessels, 1 indicating a normal caliber vessel without tumor signals, 2 indicating a mildly dilated vessel without tumor signals, 3 indicating intermediate tumor signals within a dilated vessel, and 4 indicating an irregular vessel contour or nodular tumor expansion (3). mrEMVI scores of 0–2 and 3–4 were considered negative and positive, respectively. The SyPSIR scoring criteria were as follows: 0 indicates no visible extramural vessels; 1 indicates normal caliber vessels without luminal abnormalities; 2 indicates enlarged vessels without intraluminal signal changes; 3 indicates enlarged vessels with nodular or irregular low-signal areas within the lumen; and 4 indicates markedly dilated, tortuous vessels with extensive low/intermediate signal intensity or filling defects. The modified mrEMVI scoring system incorporates the SyPSIR evaluation, with either T2-weighted imaging or SyPSIR scores of 3–4 considered to indicate positive EMVI, thereby increasing diagnostic sensitivity through the enhanced vascular visualization (Fig 2)

**Figure 1:**
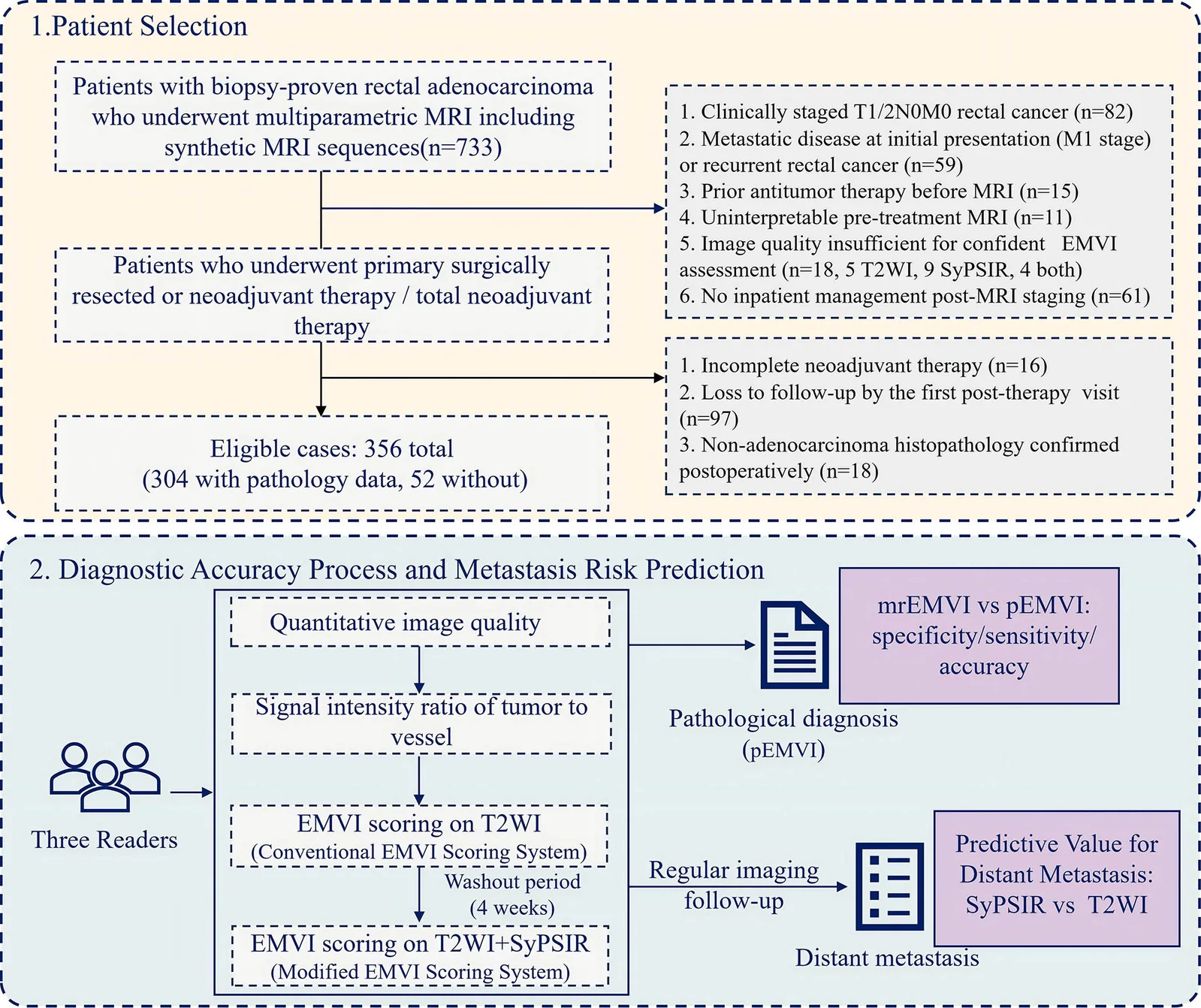
Flow charts show participant enrollment and study design protocol. EMVI = extramural vascular invasion, mrEMVI = MR-based EMVI, pEMVI = pathological extramural vascular invasion, SyPSIR = synthetic phase-sensitive inversion recovery, T2WI = T2-weighted imaging.

**Figure 2:**
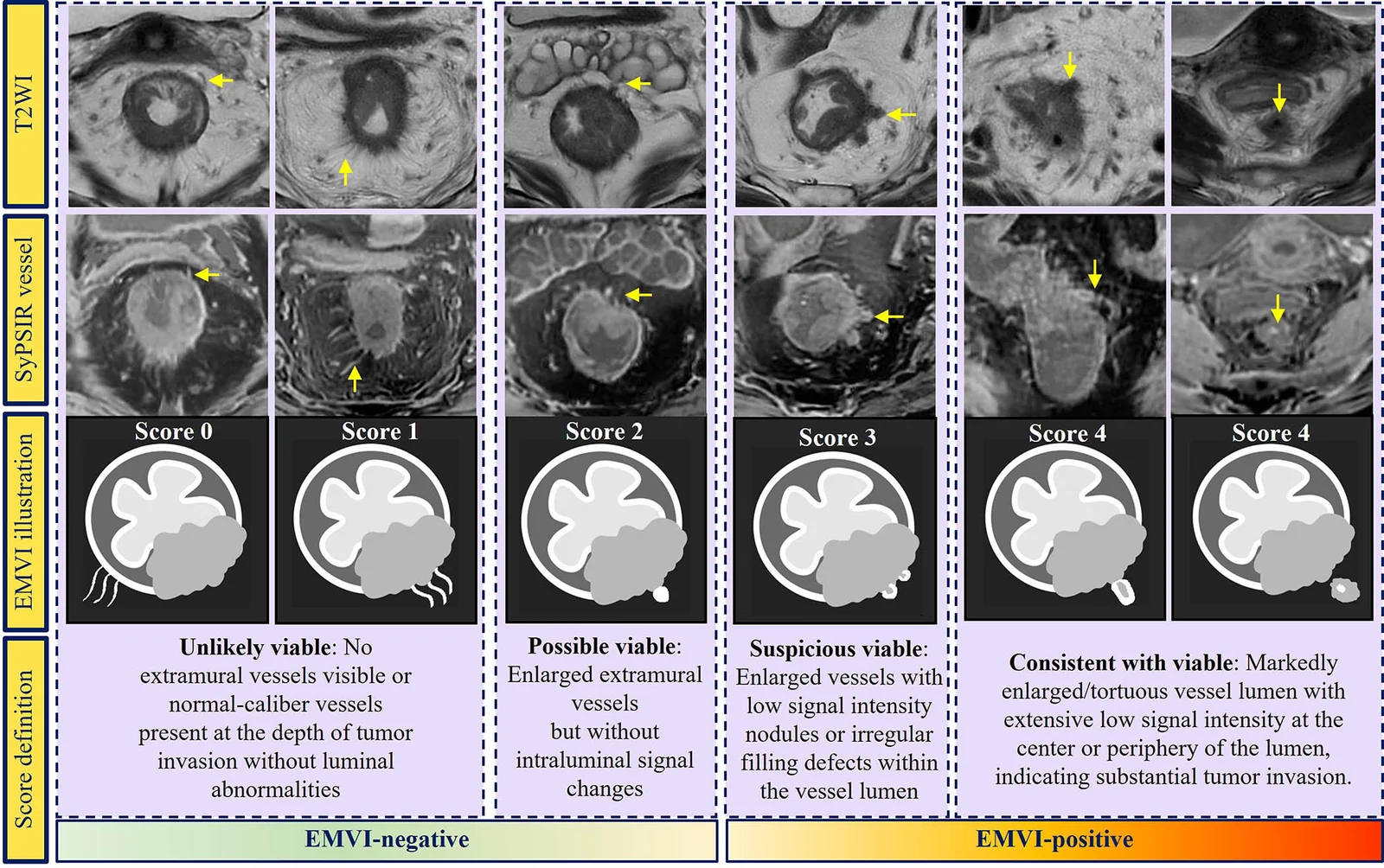
Image shows the modified MR-based extramural vascular invasion (mrEMVI) scoring system for patients with rectal cancer. SyPSIR = synthetic phase-sensitive inversion recovery, T2WI = T2-weighted imaging.

### Treatment Protocol and Pathologic Evaluation

Treatment strategies followed current guidelines with risk-adapted approaches (22,23). Among the 356 participants, 304 underwent direct surgery, whereas 52 received neoadjuvant therapy. Direct surgical decisions were made for those with emergency presentations (*n* = 74), those requiring borderline staging (*n* = 133), and those with comprehensive clinical considerations (*n* = 97). Surgery was performed within 1 week of the MRI examination. Two experienced pathologists (>10 years of experience) independently assessed venous invasion status from hematoxylin-eosin– and CD34-stained samples while blinded to the clinical and imaging data using established pathologic criteria for EMVI determination (3). The details of the pathologic evaluation are provided in Appendix S1.

### Follow-up and Outcome Assessment

Participants were followed up according to standardized surveillance protocols, with clinical evaluations every 3 months for the first 2 years, every 6 months for years 3–5, and annually thereafter. The cohort included 349 and 113 participants with complete follow-up data at 1 and 3 years posttreatment, respectively. The primary end point was metastasis-free survival (MFS), defined as the interval from the initial pathologic diagnosis to either radiologically confirmed distant metastasis or death from any cause, whichever occurred first. Among the 356 participants, 93 experienced end point events, including 91 who developed distant metastases and 2 who died without documented metastases during follow-up. Participants without events were censored at the date of the last follow-up or on June 30, 2025, whichever occurred first. Distant metastasis was independently assessed by a blinded radiologist (H.Z.) using standardized imaging criteria, with a consensus review for uncertain images. Standardized imaging criteria for hematogenous distant metastasis included: new hepatic lesions larger than 10 mm showing peripheral rim enhancement, target sign, hypovascular appearance at contrast-enhanced CT/MRI, or restricted diffusion at MRI; new pulmonary solid nodules at least 5 mm or multiple nodules with metastatic pattern at chest CT; and new osseous or cerebral lesions with typical metastatic imaging characteristics. The detailed follow-up protocols and imaging criteria are provided in Appendix S1.

### Statistical Analysis

Sample size calculation was performed based on two primary objectives. For comparing diagnostic performance between scoring systems, assuming an improvement in accuracy from 65% to 75% with 80% power and an α of 0.05, a minimum of 156 participants was required. For survival analysis, based on literature-reported independent prognostic factors in rectal cancer (mrT stage, mrN stage, mesorectal fascia status, EMVI status, and tumor deposits), we required 50 metastatic events to reliably analyze five variables using the 10 events per variable rule. Assuming a 30% 3-year metastasis rate and a 10% loss to follow-up, the minimum sample size was calculated as 185 participants.

For all analyses, binary classification was applied, with EMVI scores of 0–2 and 3–4 classified as negative and positive, respectively. Interobserver and intraobserver agreement for image quality assessment was evaluated using Fleiss κ for multiple raters and Cohen κ for repeated measures. The tumor-to-vascular signal intensity ratios were compared using paired *t* tests. For the diagnostic performance evaluation, only the 304 participants who underwent direct surgery were included to pathologically validate the results. Fifty-two participants who received neoadjuvant therapy were excluded from the diagnostic analysis because of treatment-induced pathologic changes. Diagnostic performance was assessed via receiver operating characteristic curve analysis using the DeLong test for comparisons. All 356 participants were included in the metastasis prediction analysis. The distant metastasis prediction cohort was randomly divided into training and validation sets at a 7:3 ratio, with group comparisons performed with χ^2^ or Fisher exact tests for categorical variables and Student *t* test or Mann-Whitney *U* tests for continuous variables (nonnormal data are presented as medians and IQRs). Univariable and multivariable Cox regression analyses were performed to identify independent predictors of MFS. The multivariable model included adjustment for demographic confounders (age and sex) and the primary investigated variables being imaging features (mrEMVI, mrT stage, mrN stage, mesorectal fascia status, and tumor deposits). Variables with a *P *value less than .05 in the univariable Cox regression analysis were included in the multivariable model to prevent overfitting and maintain statistical power. The prognostic model developed on the training cohort was applied to the validation cohort. Model performance was evaluated using the Harrell concordance index to assess discriminative ability. Calibration plots were used to assess the agreement between the predicted and observed probabilities at 36 months. Decision curve analysis was used to evaluate the clinical net benefit across threshold probabilities. A two-tailed *P* value less than .05 was considered to indicate statistical significance. Statistical analyses were performed (K.W.) using R (version 4.2.2; R Foundation for Statistical Computing), MSTATA (*http://www.mstata.com*), SPSS (version 24.0; IBM), and MedCalc (version 20.215; MedCalc Software).

## Results

### Demographic and Clinicopathologic Characteristics

Of the 733 participants with rectal adenocarcinoma, 82 were excluded due to clinically staged T1/2N0M0 disease, and 59 had metastatic disease at initial presentation (M1 stage) or recurrent cancer. Fifteen were excluded for prior antitumor therapy before baseline MRI, and 11 did not complete MRI examinations due to technical reasons. Image quality was insufficient for EMVI assessment in 18 cases (five with inadequate T2-weighted imaging, nine with inadequate SyPSIR, and four with inadequate quality on both sequences). Following MRI staging, 61 did not receive inpatient management. Among those who initiated treatment, 16 had incomplete neoadjuvant therapy courses, 97 were lost to follow-up within the first year after therapy, and 18 had nonadenocarcinoma histopathology confirmed postoperatively. Thus, 356 participants (mean age, 65 years ± 10 [SD]; 238 male) were included in the final analysis (Fig 1, Table 1). The median follow-up was 23 months (range, 3–50 months), with follow-up intervals of 3–6 months.

**Table 1:** Baseline Characteristics of the Study Population

Characteristic	Finding (*n* = 356)
Sex	
Male	238 (67)
Female	118 (33)
Age (y)	65 ± 10
Tumor longitudinal extent (cm)	4.17 ± 1.93
TIL (%)	68 ± 23
MR tumor location	
Lower	150 (42)
Middle	136 (38)
Upper	70 (20)
Mucinous component	
No	337 (95)
Yes	19 (5)
T stage	
T2	65 (18)
T3	169 (48)
T4	122 (34)
mrN stage	
N0	87 (24)
N1	167 (47)
N2	102 (29)
Tumor deposit	
Absent	327 (92)
Present	29 (8)
MRF involvement	
No	254 (71)
Yes	102 (29)
Treatment	
Surgery	304 (85)
Neoadjuvant therapy	30 (8)
Long-course treatment	22 (6)

Note.—Data are numbers with percentages in parentheses or means ± SDs. MRF = mesorectal fascia, TIL = tumor involvement in the circumference of the intestinal lumen.

### Interobserver Agreement

Interobserver agreement for image quality assessment between T2-weighted and SyPSIR images ranged from 0.65–0.77 across radiologists with different levels of experience, with junior radiologists demonstrating slightly lower agreement values. The quality of the SyPSIR images was good, with an intraobserver agreement κ of 0.76 and an interobserver agreement κ of 0.73. The tumor-to-vessel signal intensity ratios were markedly greater for SyPSIR images than for T2-weighted images (1.33–1.35 vs 1.03–1.22; reader 1: *P *< .001, reader 2:* P *< .001) (Table S2).

### SyPSIR Improved EMVI Detection

Pathology revealed that 76 of the 304 participants were EMVI positive, and the other 228 were EMVI negative. On SyPSIR images, pathologically positive EMVI manifested as enlarged hyperintense vessels containing intraluminal hypointense tumor signals or hypointense tumor signals adjacent to vascular structures (Fig 3). Compared with the conventional scoring system, the modified mrEMVI scoring system demonstrated better diagnostic performance (area under the receiver operating characteristic curve, 0.72–0.80 vs 0.59–0.68; *P *< .05), with notable increases in sensitivity from 33%–49% (25 of 76 to 37 of 76) to 59%–80% (45 of 76 to 61 of 76) across radiologists of all experience levels (*P* < .05) while preserving high specificity (Fig S1, Table 2). Compared with the conventional assessment, the modified system yielded better positive predictive values (41%–52% vs 33%–39%) and negative predictive values (84%–92% vs 78%–81%) (Table 2).

**Figure 3:**
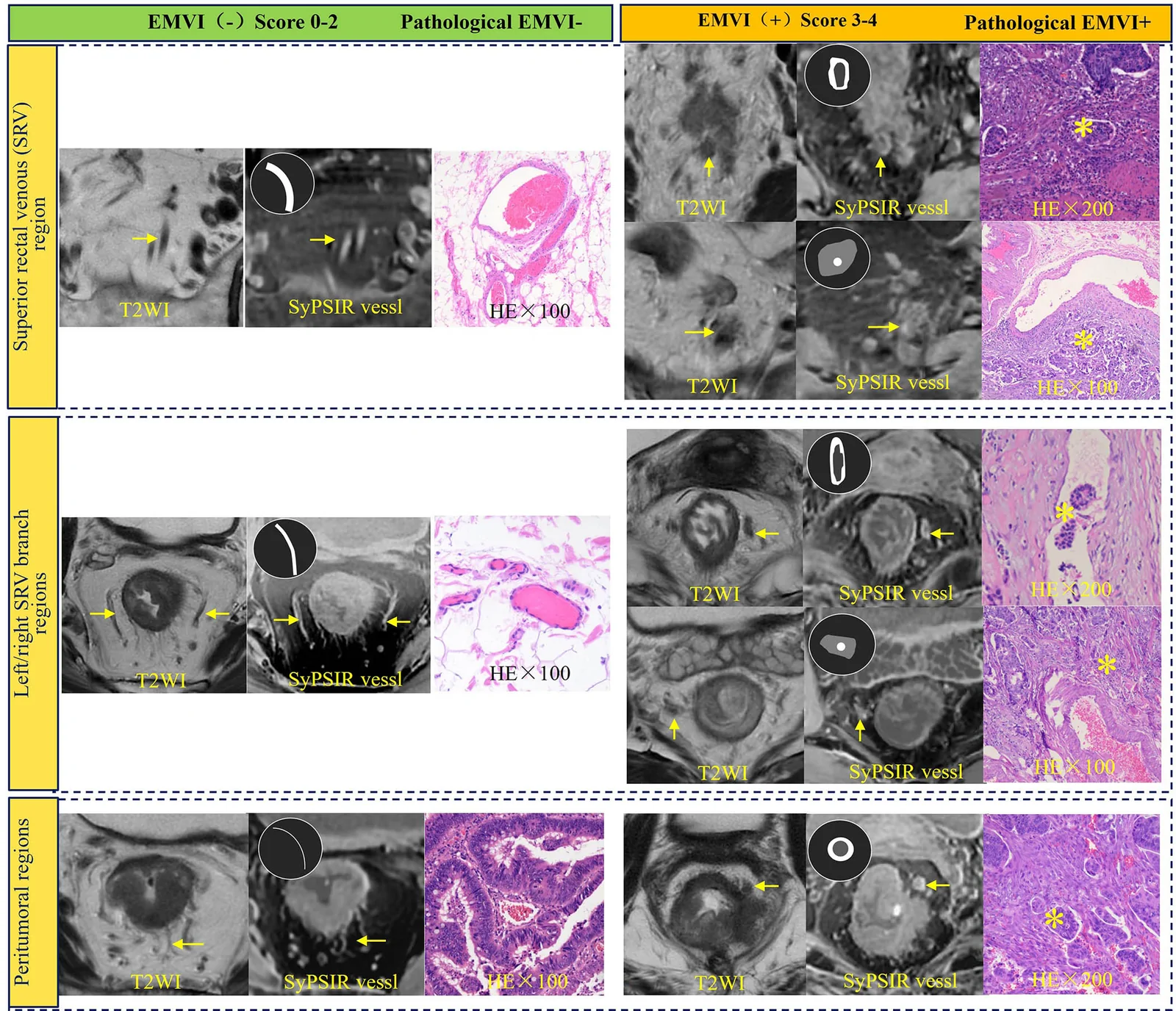
Pathologic imaging shows relationships between extramural vascular invasion (EMVI) and T2-weighted imaging (T2WI)/synthetic phase-sensitive inversion recovery (SyPSIR) in different anatomic regions. This composite figure shows EMVI-negative (scores of 0–2; left column) and EMVI-positive (scores of 3–4; right column) manifestations across the SRV, branch, and peritumoral zones. Arrows denote vessels in T2WI and SyPSIR images, regardless of EMVI status (scores 0–2 for EMVI-negative; scores 3–4 for EMVI-positive). Pathologic validation (hematoxylin-eosin staining [HE], right column) confirms the imaging findings, with yellow * indicating venous tumor thrombi corresponding to each pattern; the cellular details of complex thrombi are shown in the magnified images.

**Table 2:** Diagnostic Performance of mrEMVI for Pathologic EMVI in Patients with Rectal Cancer

Observer	EMVI Scoring System	Sensitivity (%)	Specificity (%)	Accuracy (%)	PPV (%)	NPV (%)	AUC (95% CI)	DeLong Test	
								Z	*P* Value
Reader 1 (senior)	SyPSIR and T2WI	80 (61/76)	75 (171/228)	76 (232/304)	51 (61/118)	91 (171/186)	0.80 (0.75, 0.84)	4.87	<.0001
	T2WI	49 (37/76)	74 (169/228)	68 (206/304)	39 (37/96)	81 (169/208)	0.68 (0.62, 0.73)		
Reader 2 (midcareer)	SyPSIR and T2WI	72 (55/76)	74 (171/228)	74 (226/304)	49 (55/112)	89 (171/192)	0.76 (0.71, 0.81)	3.563	.0004
	T2WI	42 (32/76)	75 (174/228)	68 (206/304)	37 (32/86)	79 (174/218)	0.66 (0.60, 0.71)		
Reader 3 (junior)	SyPSIR and T2WI	59 (45/76)	71 (163/228)	68 (208/304)	41 (45/110)	84 (163/194)	0.72 (0.66, 0.77)	3.378	.0007
	T2WI	32 (25/76)	78 (178/228)	67 (203/304)	33 (25/75)	78 (178/229)	0.59 (0.53, 0.65)		

Note.— AUC = area under the receiver operating characteristic curve, EMVI = extramural vascular invasion, mrEMVI = MR-based EMVI, NPV = negative predictive value, PPV = positive predictive value, SyPSIR = synthetic phase-sensitive inversion recovery, T2WI = T2-weighted imaging.

### Relationships between mrEMVI Status and Metastasis-Free Survival

Cox regression analyses were conducted to establish the modified mrEMVI scoring system as an independent predictor of distant metastasis, using 249 participants for model development and 107 participants for internal validation, with balanced clinical and imaging characteristics between cohorts (Table S3). Univariable Cox analysis revealed the modified mrEMVI score as a potent predictor of MFS (hazard ratio, 13.93; *P* < .001). Four other variables—the mucinous component, mrT stage, mrN stage, and tumor deposits—were also identified as predictors. Multivariable analysis confirmed that the modified mrEMVI score was an independent prognostic factor (hazard ratio, 10.76; 95% CI: 5.09, 22.74; *P* < .001), in addition to the mucinous component (hazard ratio, 2.63; 95% CI: 1.25, 5.56; *P* = .011) (Table 3). Kaplan-Meier survival curves confirmed the superior risk stratification performance of the modified mrEMVI system, with participants who are EMVI positive showing worse MFS than EMVI-negative participants did in both the training and validation cohorts (all *P* < .001) (Fig 4).

**Table 3:** Univariable and Multivariable Cox Analyses for Rectal Cancer Distant Metastasis in the Training Cohort

Characteristic	N	No. of Events	Univariable Analysis		Multivariable Analysis	
			HR (95% CI)	*P* Value	HR (95% CI)	*P* Value
Sex						
Male	170	45	…	.75	…	…
Female	79	23	1.08 (0.66, 1.79)		…	…
Age (y)	249	68	1.00 (0.98, 1.02)	.95	…	…
Mesorectal tumor location						
Lower	99	24	…	.57	…	…
Middle	97	26	1.18 (0.67, 2.05)	.22	…	…
Upper	53	18	1.46 (0.79, 2.70)		…	…
Mucus						
No	234	59	…	.002	…	…
Yes	15	9	3.07 (1.52, 6.21)		2.63 (1.25, 5.56)	.01
Tumor longitudinal extent (cm)	249	68	1.08 (0.94, 1.25)	.27	…	…
TIL (%)	249	68	2.38 (0.84, 6.74)	.10	…	…
mrT stage						
2	47	2	…	…	…	…
3	117	31	7.13 (1.71, 29.80)	.007	4.49 (0.98, 20.51)	.053
4	85	35	13.82 (3.31, 57.66)	<.001	4.32 (0.98, 21.37)	.053
mrN stage						
0	59	7	…	…	…	…
1	124	33	2.52 (1.12, 5.71)	.03	1.62 (0.67, 3.94)	.29
2	66	28	4.76 (2.07, 10.91)	<.001	1.95 (0.78, 4.88)	.15
Tumor deposit						
No	222	51	…	<.001	…	…
Yes	27	17	3.81 (2.19, 6.62)		1.16 (0.64, 2.11)	.62
MRF						
No	176	43	…	.09	…	…
Yes	73	25	1.54 (0.94, 2.52)	…	…	…
Treatment						
Surgery	216	58	…	…	…	…
Neoadjuvant therapy	20	6	1.04 (0.45, 2.42)	.92	…	…
Long-course treatment	13	4	1.58 (0.57, 4.36)	.38	…	…
Modified EMVI Assessment						
Negative	141	9	…	<.001	…	…
Positive	108	59	13.93 (6.87, 28.23)		10.76 (5.09, 22.74)	<.001

Note.—EMVI = extramural vascular invasion, HR = hazard ratio, MRF = mesorectal fascia, TIL = tumor involvement in the circumference of the intestinal lumen.

**Figure 4:**
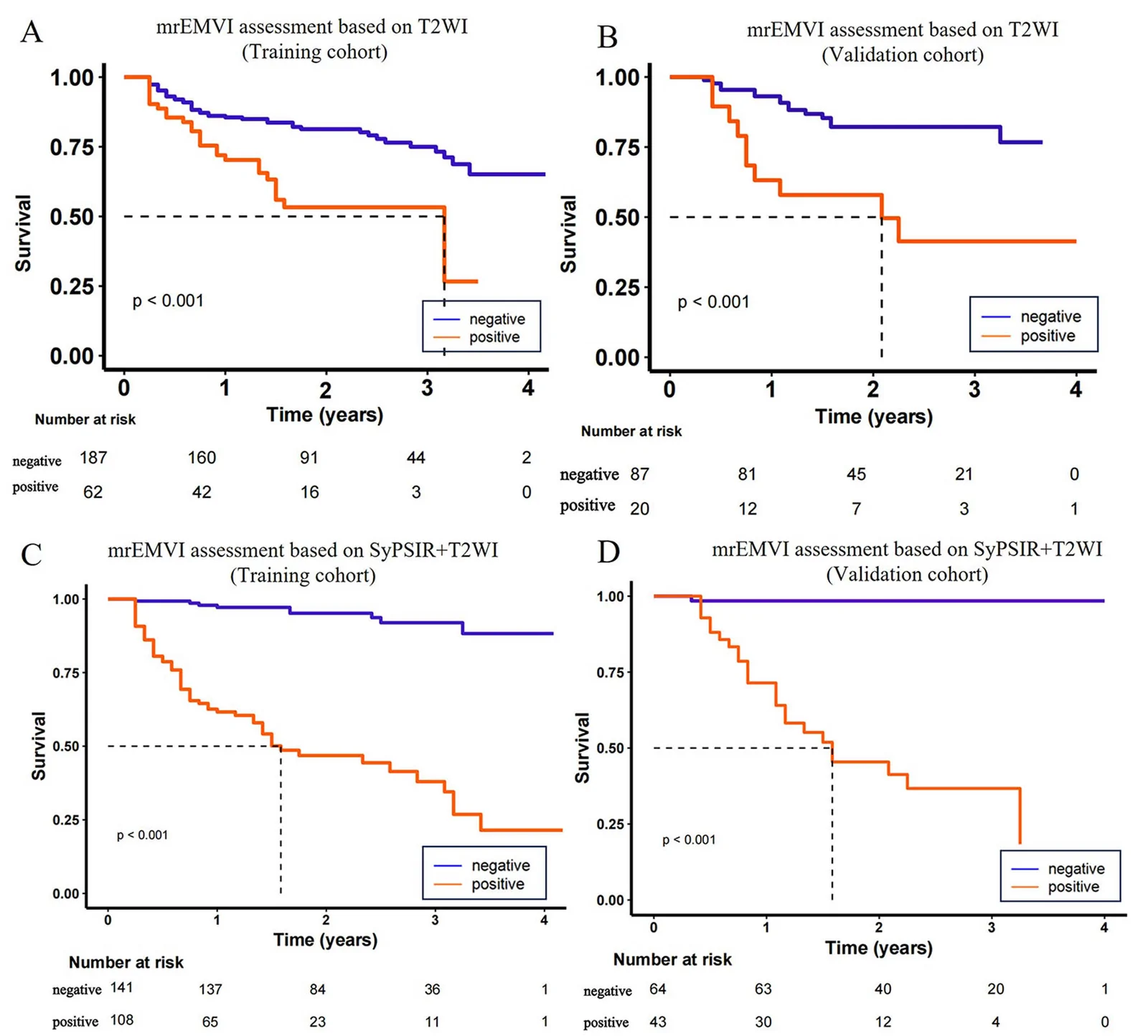
Kaplan-Meier metastasis-free survival curves stratified by MR- based extramural vascular invasion (mrEMVI) status using conventional T2-weighted imaging (T2WI) versus the modified synthetic phase-sensitive inversion recovery (SyPSIR) and T2WI scoring system. **(A)** Training cohort assessed using conventional T2WI (*n* = 249; EMVI negative: *n* = 187, EMVI positive: *n* = 62). **(B)** Validation cohort assessed using conventional T2WI (*n* = 107; EMVI negative: *n* = 87, EMVI positive: *n* = 20). **(C)** Training cohort assessed using the modified SyPSIR and T2WI scoring system (*n* = 249; EMVI negative: *n* = 141, EMVI positive: *n* = 108). **(D)** Validation cohort assessed using the modified SyPSIR and T2WI scoring system (*n* = 107; EMVI negative: *n* = 64, EMVI positive: *n* = 43). Blue lines represent patients who are EMVI negative; orange lines represent patients who are EMVI positive. *P* values shown were calculated using the log-rank test.

### Comparison of Modified and Conventional mrEMVI Systems

To validate the clinical utility of the modified mrEMVI scoring system, a head-to-head comparison was performed with the conventional scoring system to evaluate their respective abilities to stratify patients according to metastasis risk. In the training cohort, the modified system achieved concordance index values of 0.77–0.82 across the three readers, compared with 0.75–0.76 for the conventional system. These improvements were consistent in the validation cohort (modified, 0.80–0.88 vs conventional, 0.70–0.76). The 36-month time-dependent area under the receiver operating characteristic curve values in the validation cohort also favored the modified system (0.86–0.98) over the conventional system (0.76–0.79) (Table 4). On the basis of its better performance, the senior radiologist’s (H.Z.) modified mrEMVI assessment was selected to construct a nomogram.

**Table 4:** Comparison of Modified and Conventional mrEMVI Classification Models for Prognosis Stratification of Patients with Rectal Cancer with Respect to MFS

Observer	EMVI Classification Models	Training Cohort (*n* = 249)			Internal Test Cohort (*n* = 107)	
		EMVI and Status HR (95% CI)	Concordance Index (95% CI)	AUC for 36-Month MFS (95% CI)	Concordance Index (95% CI)	AUC for 36-Month MFS (95% CI)
Reader 1	Modified	10.76 (5.09, 22.74)	0.82 (0.78, 0.87)	0.86 (0.81, 0.96)	0.88 (0.83, 0.93)	0.98 (0.95, 1.01)
	Conventional	1.51 (0.86, 2.66)	0.76 (0.71, 0.81)	0.84 (0.75, 0.92)	0.76 (0.67, 0.86)	0.79 (0.63, 0.94)
Reader 2	Modified	5.83 (3.11, 10.92)	0.80 (0.76, 0.85)	0.87 (0.79, 0.95)	0.84 (0.76, 0.91)	0.91 (0.81, 1.00)
	Conventional	1.26 (0.71, 2.24)	0.76 (0.70, 0.81)	0.82 (0.73, 0.91)	0.73 (0.64, 0.83)	0.78 (0.62, 0.93)
Reader 3	Modified	2.88 (1.65, 5.04)	0.77 (0.72, 0.82)	0.83 (0.75, 0.92)	0.80 (0.71, 0.88)	0.86 (0.72, 1.00)
	Conventional	0.94 (0.53, 1.65)	0.75 (0.69, 0.81)	0.80 (0.71, 0.90)	0.70 (0.60, 0.80)	0.76 (0.61, 0.92)

Note.—AUC = area under the receiver operating characteristic curve, EMVI = extramural vascular invasion, HR = hazard ratio, MFS = metastasis-free survival, mrEMVI = MR-based EMVI.

### Development and Validation of the Prognostic Nomogram

On the basis of the independent prognostic factors (modified mrEMVI, mucinous component, mrN stage, and mrT stage) confirmed by the multivariable analysis, a clinical nomogram was constructed to quantify individualized metastasis risk stratification (Fig 5A). The model demonstrated excellent predictive performance, achieving 36-month time-dependent areas under the receiver operating characteristic curve of 0.86 and 0.98 with the training and validation cohorts (Fig 5B–C), confirming its robust clinical utility for metastasis risk stratification. The calibration curve closely approximated the diagonal line in the training cohort (Brier score, 11.5; 95% CI: 7.4, 15.5), indicating good agreement between predicted and observed probabilities (Fig 5D). The validation cohort showed acceptable calibration (Brier score, 6.9; 95% CI: 2.9, 10.8) with some deviation in the intermediate risk range (Fig 5E). Decision curve analysis confirmed the superior net clinical benefit of the nomogram across relevant threshold probabilities compared with treating all or no patients (Fig 5F–G). These findings demonstrate that incorporating SyPSIR sequences into mrEMVI assessment substantially improves prognostic stratification in patients with locally advanced rectal adenocarcinoma.

**Figure 5:**
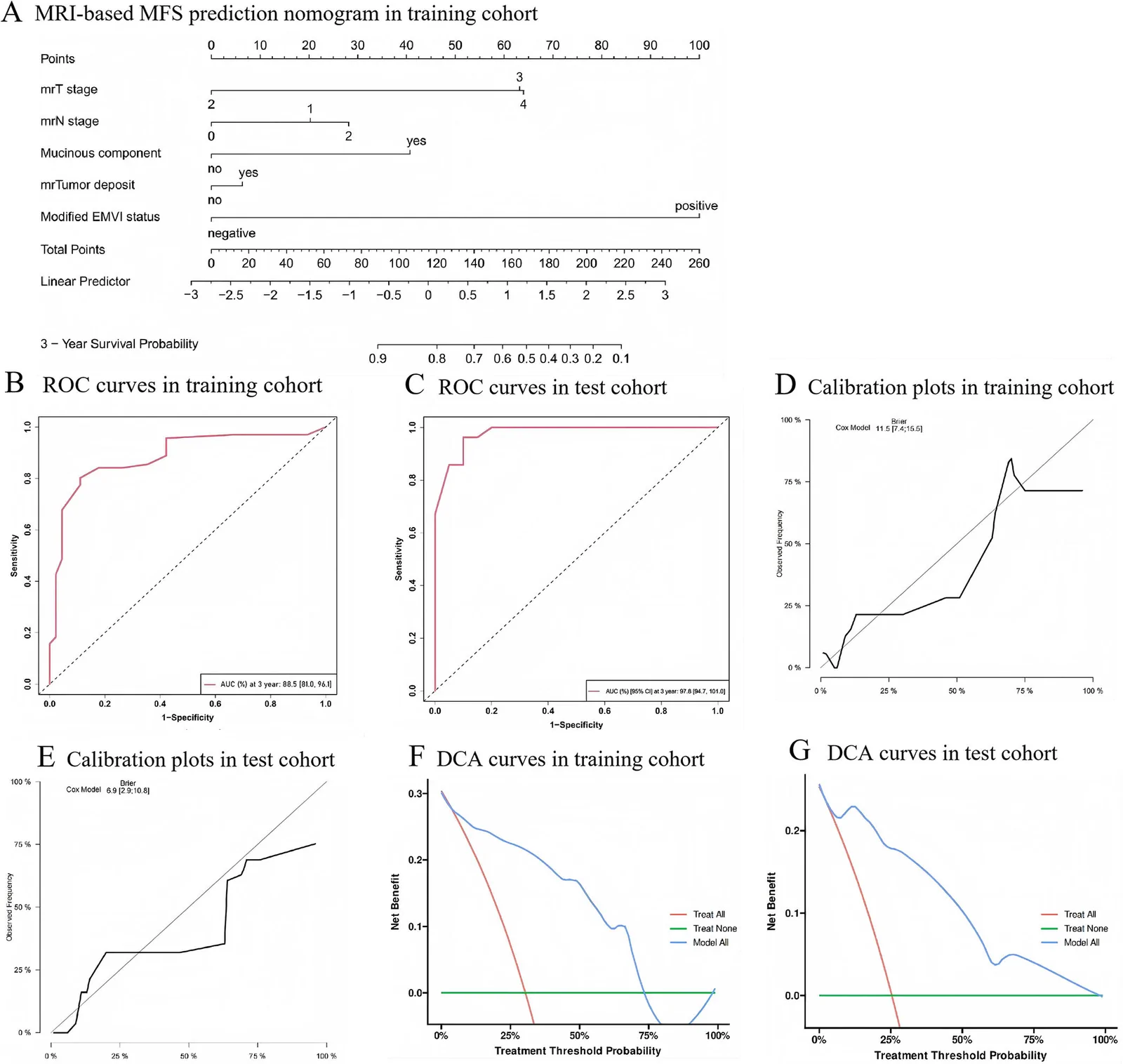
Development and validation of a multiparametric MRI-based nomogram for metastasis-free survival (MFS) prediction in patients with locally advanced rectal cancer. **(A)** Nomogram incorporating the modified MR-based extramural vascular invasion (mrEMVI) score, mrT stage, mrN stage, mucinous component, and tumor deposits based on the synthetic phase-sensitive inversion recovery (SyPSIR) and T2-weighted imaging (T2WI) scoring system (scale: EMVI status contributes 50% weight) for predicting 36-month MFS probabilities. **(B)** Receiver operating characteristic (ROC) curves for 36-month MFS prediction in the training cohort. **(C)** ROC curves for 36-month MFS prediction in the validation cohort. **(D)** Calibration plot for 36-month MFS prediction in the training cohort. **(E)** Calibration plot for 36-month MFS prediction in the validation cohort. **(F)** Decision curve analysis (DCA) for 36-month MFS prediction in the training cohort. **(G)** DCA for 36-month MFS prediction in the validation cohort. AUC = area under the receiver operating characteristic curve.

## Discussion

The conventional T2-weighted imaging–based mrEMVI scoring system suffers from suboptimal diagnostic performance and unreliable risk stratification for patients with rectal cancer. This study presented a modified mrEMVI scoring system that integrated SyPSIR image data. Compared with the conventional mrEMVI scoring system, the modified system showed markedly better vascular invasion detection (sensitivity: 59%–80% vs 33%–49%; area under the receiver operating characteristic curve, 0.72–0.80 vs 0.59–0.68; *P* < .05). For metastasis prediction, the modified mrEMVI demonstrated superior prognostic performance as the strongest independent predictor of MFS (hazard ratio, 10.76 vs 5.46; *P* < .001), with the integrated nomogram achieving excellent discrimination for 3-year MFS (area under the receiver operating characteristic curve, 0.86–0.98). Given that current guidelines stratify patients who are mrEMVI positive into high-risk categories requiring intensive treatments like chemoradiotherapy or total neoadjuvant therapy (22,23), the enhanced accuracy of the SyPSIR-based approach could fundamentally improve patient management through more precise risk stratification and individualized treatment selection.

The modified EMVI scoring system incorporating SyPSIR images achieved a diagnostic sensitivity of 59%–80% across radiologists with different experience levels, outperforming both the conventional scoring system (33%–49%) and existing advanced approaches such as diffusion-weighted imaging–based techniques, dynamic contrast-enhanced MRI, and deep learning algorithms (55%–68%) (13–17). Importantly, this greater sensitivity was accompanied by an increase in the positive predictive value from 33%–39% to 41%–52%, indicating that the additional detected images were predominantly true positives rather than false positives. By inverting vessel signal to hyperintense while preserving tumor deposits as hypointense filling defects, SyPSIR enhances tumor–vessel contrast, enabling detection of an additional 26%–31% of EMVI cases missed on conventional imaging. This technical advance provides a practical solution to the long-standing diagnostic challenge that has limited accurate preoperative EMVI assessment in clinical practice.

Previous studies have consistently identified mrEMVI as an independent prognostic factor in rectal cancer. Van den Broek et al (24) demonstrated a fourfold increased risk of metachronous metastases in patients who are mrEMVI positive. A meta-analysis by Rouleau Fournier et al (25) comprising 3821 patients reported a pooled hazard ratio of 2.30 for disease recurrence in patients who are preneoadjuvant mrEMVI positive. Consistent with these findings, our analysis confirmed mrEMVI as an independent predictor of MFS in locally advanced rectal cancer. The hazard ratios observed in our cohort exceeded those reported in previous meta-analyses (25). This enhanced prognostic discrimination likely reflects more comprehensive capture of truly EMVI-positive cases through the modified scoring system incorporating SyPSIR sequences. Notably, the modified system showed higher performance in the validation cohort than the training cohort, a pattern not observed with conventional scoring. This paradoxical improvement likely reflects case mix differences, with validation cases potentially demonstrating more prominent SyPSIR-enhanced features. Despite this limitation, the modified system maintained consistent performance across radiologists with varying expertise levels, validating its reproducibility. Thus, the SyPSIR-based approach provides superior prognostic stratification with robust clinical applicability.

Beyond mrEMVI, our multivariable analysis revealed that MRI-based T and N staging lost independent prognostic significance for MFS, consistent with Djuric et al (26) who demonstrated superior prognostic performance of EMVI over pathologic T and N staging and lymphovascular invasion. Previous studies have noted that the current TNM system inadequately recognizes alternative dissemination routes, particularly the direct vascular pathway affecting 40% of patients with rectal cancer (27). This suggests that venous channels provide an anatomic highway for rapid tumor dissemination to distant sites, bypassing lymphatic pathways entirely. Through this venous route, Li et al (28) demonstrated significantly higher circulating tumor cell detection in first drainage vein blood versus peripheral blood, with circulating tumor cell levels correlating with liver metastasis development. These circulating tumor cells traveling through venous pathways identified by mrEMVI represent cellular mediators of distant metastasis. Thus, mrEMVI indicates not merely local vascular involvement but also active hematogenous dissemination, explaining its superior prognostic discrimination over traditional staging parameters that primarily reflect local tumor extent.

This study had several limitations. First, 97 patients were excluded because of loss to follow-up within the first posttherapy period. This could introduce selection bias and limit the generalizability of the results to a wider clinical population. Second, delayed metastatic events may not have been captured owing to the relatively brief median follow-up of 23 months, potentially affecting the accuracy of the survival analysis and long-term prognostic assessment. Third, patients who received neoadjuvant therapy (*n* = 52) and those who underwent direct surgery (*n* = 304) were combined in the analysis, which represents a potential confounder because neoadjuvant treatment can alter imaging characteristics. Fourth, a single MRI vendor (GE HealthCare) and specific commercial software (MAGiC, Synthetic MR) were used, which may limit the generalizability of the findings to institutions using different equipment, despite the cross-platform compatibility of the proposed technique (19). Finally, the subjective qualitative descriptors used in the scoring system may introduce interreader variability despite the good agreement among the experienced radiologists. External validation through multicenter studies across diverse imaging platforms is needed to confirm the reproducibility of the findings and the broader clinical applicability of the proposed approach.

In conclusion, the results suggest that the modified mrEMVI scoring system incorporating SyPSIR image data may improve the preoperative detection of vascular invasion and could contribute to refined distant metastasis risk stratification, potentially serving as a prognostic indicator for MFS. Because SyPSIR generates contrast-like vascular signal intensity without gadolinium-based contrast material administration, this technique represents a step toward contrast-free, high-resolution vascular assessment in rectal cancer staging, with potential integration into routine MRI protocols for participants ineligible for contrast-enhanced examination. Future studies should elucidate the prognostic significance of EMVI anatomic distribution and morphologic characteristics in relation to metastatic outcomes and evaluate the applicability of this scoring system in postneoadjuvant therapy reassessment.

## Supplemental Files

Appendix S1, Tables S1-S3, Figure S1

Conflicts of Interest
