# Supplementary material for: Synthetic Phase-Sensitive Inversion Recovery and Extramural Vascular Invasion Scoring Systems for Vascular Invasion Detection and Metastasis Risk in Rectal Cancer
Source: Radiol Imaging Cancer. 2026 Sep 18;8(5):e250564. doi: 10.1148/rycan.250564 (PMC13620328; doi:10.1148/rycan.250564)

Supplemental materials undergo peer review but are not copyedited, and may include typographical errors.

©RSNA, 2026
10.1148/rycan.250564

Appendix S1

Image quality assessment

Three radiologists with varying levels of experience (5-22 years of gastrointestinal tumor diagnostic experience) independently analyzed the quality of T2WI and SyPSIR sequences. All reviewers were blinded to the study design, clinical patient data, diagnoses, and final pathology reports. Overall image quality scores (1= poor image quality, 5= excellent quality, optimal tumor contrast) were assessed using a 5-point Likert scale.

Signal intensity analysis

Two experienced radiologists (senior and intermediat) measured signal intensities (SI) of tumor tissue and normal superior rectal venous (SRV) on both T2WI and SyPSIR sequences. The relative contrast between tumor and vessel ($SI_{tumor-vessel}$) was calculated using the following equation (17):

$SI_{tumor-vessel} = |(SI_{tumor} - SI_{vessel})/(SD_{tumor^2} + SD_{vessel^2})^{\wedge}1/2|$

Tumor regions of interest (ROI) were manually delineated along the edge of the largest axial tumor section, avoiding necrosis, mucin, and bowel contents. Vessel ROIs were drawn along the normal superior rectal venous contour, with all ROIs required to exceed 10 mm^2 .

Treatment protocol and pathologic evaluation

The interval between MRI examination and surgery was within one week for all 304 patients, who underwent various surgical procedures (Dixon, ultra-low anterior resection, intersphincteric resection, Miles, and Hartmann procedures). Two experienced pathologists (each with more than 10 years of rectal cancer pathology experience) independently assessed the venous invasion status from hematoxylin-eosin (HE) and CD34-stained surgical specimens while remaining blinded to all clinical and imaging data. Venous invasion is defined as the presence of tumor cells within endothelium-lined vascular spaces that are either surrounded by a smooth muscle wall or contain fibrin and red blood cells. Modern pathological criteria have expanded this definition to include tumor deposits with convincing elastin staining around their periphery, forming rounded or elongated profiles typically found adjacent to vascular structures. The EMVI status was recorded as present and absent. Any discrepancies or ambiguities in the reports prompted discussions between the pathologists, and when consensus could not be reached, a third senior pathologist was consulted for final determination.

Follow-up protocols and imaging criteria

For metastatic events, time-to-metastasis (TTM) was calculated from the date of initial diagnosis to the first radiographic evidence of distant disease. Distant metastasis during follow-up was independently assessed by a fourth radiologist (H.Z., with 8 years of experience in abdominal imaging) who was blinded to the initial MRI interpretations and patient clinical information. Liver, lung, bone, and brain metastases were considered hematogenous distant metastases. Lymph node

Supplemental materials undergo peer review but are not copyedited, and may include typographical errors.

metastases and peritoneal carcinomatosis were excluded from the primary endpoint analysis as these represent non-hematogenous spread patterns. In cases of diagnostic uncertainty, consensus review was performed between the follow-up radiologist and a senior radiologist (W.C., with 15 years of experience), with final determination based on multidisciplinary team discussion including clinical and additional imaging findings when available.

Supplemental materials undergo peer review but are not copyedited, and may include typographical errors.

Table S1: MRI parameters

Parameter	T1WI	T2WI	DWI	Synthetic MRI
Sequence	FSE	FSE	MUSE	QRAPMASTER
Imaging plane	Axial	Oblique axial, oblique coronal, sagittal	Axial	Oblique axial
Repetition time (ms)	771	3227–5326	5383	4000
Echo time (ms)	Min full	102	Minimum	19.5/97.3
Slice thickness/gap (mm)	4/1.0	3/0.3	4/1.0	3/0.3
Field of view (cm)	38 × 38	18 × 18, 24 × 24, 24 × 24	30 × 30	24 × 24
Matrix	320 × 256	320 × 224, 320 × 256, 320 × 224	140 × 144	320× 244
Echo train length	3	24, 32, 24	–	16
Bandwidth (kHz)	41.67	83.33, 62.50, 62.50	250	27.78
Acquisition time (min:s)	1:28	2:45, 2:40, 3:06	2:41	4:32

Supplemental materials undergo peer review but are not copyedited, and may include typographical errors.

Table S2: Comparison of image quality and contrast-to-signal ratio

Parameter	T2WI	SyPSIR	*Agreement (κ) or Inter-sequence Comparison (P value)*
Overall image quality (mean±SD)			
Reader 1	4.7 ± 0.5	4.6 ± 0.5	0.70
Reader 1(repeated measures)	4.7 ± 0.5	4.6 ± 0.5	0.73
Reader 2	4.6 ± 0.5	4.7± 0.5	0.77
Reader 3	4.7 ± 0.5	4.6± 0.6	0.65
Inter-rater agreement (Fleiss' κ, 3 raters)	0.83	0.73	
Intra-rater agreement (Cohen's κ, repeated measures)	0.91	0.76	
Tumor-to-vessel signal intensity ratios (mean±SD)			
Reader 1	1.2 ± 0.2	1.3 ± 0.2	<0.001
Reader 2	1.0 ± 0.3	1.4 ± 0.1	<0.001

SI=signal intensity

Supplemental materials undergo peer review but are not copyedited, and may include typographical errors.

Table S3: Patient demographics and baseline characteristics in cox regression analysis for rectal cancer distant metastasis

Characteristic	Training Cohort N = 249	Internal Test Cohort N = 107	*P* value
Gender, n (%)			0.386
Male	170 (68.3%)	68 (63.6%)	
Female	79 (31.7%)	39 (36.4%)	
Age, Mean ± SD	65 ± 10	64 ± 10	0.281
MR tumor location, n (%)			0.310
Lower	99 (39.8%)	51 (47.7%)	
Middle	97 (39.0%)	39 (36.4%)	
Upper	53 (21.3%)	17 (15.9%)	
Mucinous component, n (%)			0.379
No	234 (94.0%)	103 (96.3%)	
Yes	15 (6.0%)	4 (3.7%)	
Tumor longitudinal extent (cm)	4.22 ± 1.84	4.06 ± 2.14	0.490
TIL (%)	67 ± 23	69 ± 25	0.594
mrT, n (%)			0.897
2	47 (18.9%)	18 (16.8%)	
3	117 (47.0%)	52 (48.6%)	
4	85 (34.1%)	37 (34.6%)	
mrN, n (%)			0.224
0	59 (23.7%)	28 (26.2%)	
1	124 (49.8%)	43 (40.2%)	
2	66 (26.5%)	36 (33.6%)	
MRF, n (%)			0.672
No	176 (70.7%)	78 (72.9%)	
Yes	73 (29.3%)	29 (27.1%)	
mrTumor deposit, n (%)			0.005
No	222 (89.2%)	105 (98.1%)	
Yes	27 (10.8%)	2 (1.9%)	
Modified EMVI status (by Senior#R1), n (%)			0.577
Negative	141 (56.6%)	64 (59.8%)	
Positive	108 (43.4%)	43 (40.2%)	
Conventional EMVI status (by Junior#R1), n (%)			0.202
Negative	187 (75.1%)	87 (81.3%)	
Positive	62 (24.9%)	20 (18.7%)	
Treatment			0.533
Surgery	216 (86.7%)	88 (82.2%)	
Neoadjuvant therapy	20 (8.0%)	11(10.3%)	
Long-course treatment	13 (5.3%)	8 (7.5%)	

Supplemental materials undergo peer review but are not copyedited, and may include typographical errors.

TIL = Tumor involvement in the circumference of intestinal lumen; TME= Total mesorectal excision.

Supplemental materials undergo peer review but are not copyedited, and may include typographical errors.

Figure S1: Diagnostic performance comparison of conventional versus modified EMVI scoring for pathologic EMVI assessment

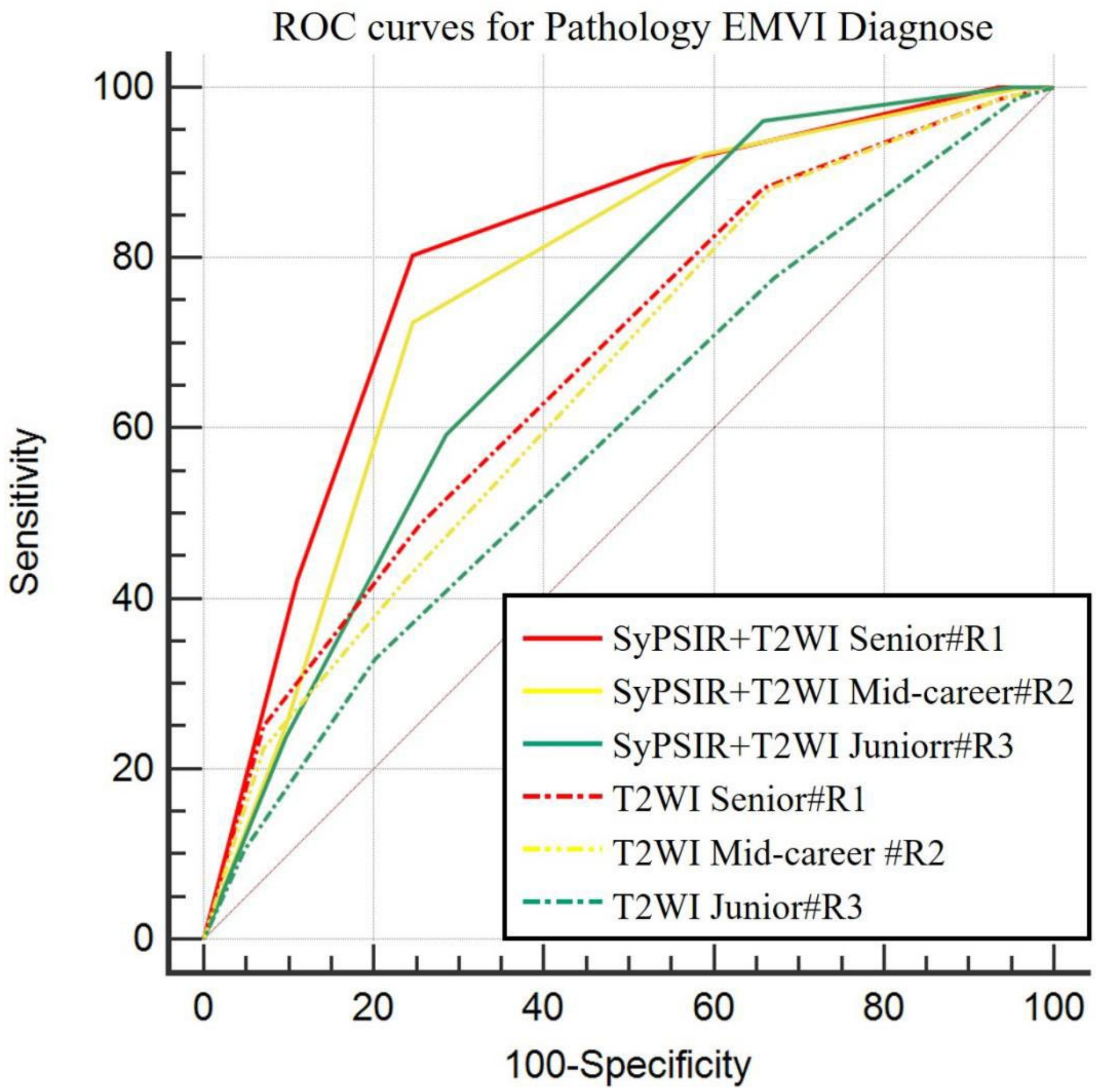

Supplement: Supplementary file 1 [file rycan250564supp.pdf]
